# Identification of regulator gene and pathway in myocardial ischemia-reperfusion injury: a bioinformatics and biological validation study

**DOI:** 10.1186/s41065-025-00397-5

**Published:** 2025-03-11

**Authors:** Yanqi Liu, Xiaodong Sheng, Zhenghong Zhao, Hongxia Li, Jiahui Lu, Lihuan Xie, Guanqun Zheng, Tingbo Jiang

**Affiliations:** 1 Department of Cardiology, The First Affiliated Hospital of Soochow University, Suzhou, Jiangsu China; 2 Department of Cardiology, The Second People’s Hospital of Changshu, Affiliated Changshu Hospital of Nantong University, Changshu, Suzhou, Jiangsu China

**Keywords:** S100A9, Inflammatory response, MIRI, CNR

## Abstract

**Background:**

Acute myocardial infarction (AMI) is the primary cause of cardiac mortality worldwide. However, myocardial ischemia-reperfusion injury (MIRI) following reperfusion therapy is common in AMI, causing myocardial damage and affecting the patient’s prognosis. Presently, there are no effective treatments available for MIRI.

**Methods:**

We performed a comprehensive bioinformatics analysis using three GEO datasets on differentially expressed genes, including gene ontology (GO), pathway enrichment analyses, and protein-protein interaction (PPI) network analysis. Cytoscape and LASSO methods were employed to identify novel regulator genes for ischemia-reperfusion (I/R). Notably, gene S100A9 was identified as a potential regulator of I/R. Additionally, clinical sample datasets were analyzed to prove the expression and mechanism of S100A9 and its down genes in I/R. The correlation of S100A9 with cardiac events was also examined to enhance the reliability of our results.

**Results:**

We identified 135 differential genes between the peripheral blood of 47 controls and 92 I/R patients. S100A9 was distinguished as a novel regulator gene of I/R with diagnostic potential. RT-qPCR test demonstrated significant upregulation of S100A9 in I/R. We also verified that S100A9 expression strongly correlates with left ventricular ejection fraction (LVEF) and MIRI.

**Conclusion:**

This study confirms that S100A9 is a key regulator of I/R progression and may participate in ischemia-reperfusion injury by upregulating RAGE /NFKB-NLRP3 activation. Elevated S100A9 levels may serve as a marker for identifying high-risk MIRI patients, especially those with coronary artery no-reflow (CNR), who might benefit from targeted therapeutic interventions. Furthermore, Peripheral blood S100A9 in AMI represents a new therapeutic target for preventing MIRI.

## Introduction

Ischemic heart disease is the primary cause of age-standardized mortality globally, significantly contributing to cardiovascular disease-related deaths [1]. While the mortality rate for acute myocardial infarction (AMI) has shown promising declines in recent years due to successful early revascularization, it remains a major health challenge, with reperfusion causing additional cardiac injury. Reperfusion following ischemia may lead to exacerbated myocardial damage, a phenomenon known as myocardial ischemia-reperfusion injury (MIRI). MIRI is a biphasic and complex pathophysiological event that occurs when blood supply is reperfusion after a period of ischemia, leading to arrhythmias and cardiac dysfunction through the generation of reactive oxygen species (ROS) [2], calcium overload [3], and inflammatory cascades [4]. This ultimately results in apoptosis, necrosis, ferroptosis, and pyroptosis [5]. Studies have shown that the damage caused by reperfusion is more severe than that caused by ischemia alone [6]. Despite the focus on immediate revascularization in clinical treatments, effective pharmacological interventions for MIRI remain lacking.

Cardiac repair following ischemia/reperfusion (I/R) injury is a highly coordinated and complex process, typically described in three intersecting phases: the inflammatory phase, characterized by immune cell infiltration and the production of danger-associated molecular patterns (DAMPs); the proliferative phase; and the maturation phase [7]. Activation of innate immune pathways through DAMPs (e.g. S100 family proteins, mobility group box 1, heat-shock proteins) and the complement cascade, recruits inflammatory leucocytes to clear the dead cells and matrix fragments from the wound [8]. The initial inflammatory response triggered by DAMPs activates pattern recognition receptors (PRRs), which release cytokines and initiate tissue repair. However, persistent or excessive inflammation following AMI would lead to ventricular dilatation, systolic dysfunction, and adverse myocardial remodeling. Accordingly, a thorough understanding of the molecular mechanisms involved in myocardial ischemia-reperfusion injury is essential for governing immune activation and improving patient outcomes.

In this study, we used three microarray datasets of I/R retrieved from the GEO datasets for integrative bioinformatics to identify I/R-related gene signatures and their functional pathways. After identifying differentially expressed genes (DEGs) through Surrogate Variable Analysis (SVA), we identified S100A9 as a potential regulator through Cytohobba and the LASSO algorithm. This finding was subsequently validated with an additional dataset.

S100A9 [9], also known as myeloid-related protein14 (MRP-14), is a calcium-zinc binding protein consisting of 114 amino acids, accounting for 40% of neutrophil cytoplasmic proteins. S100A9 non-covalently binds with S100A8 to form a 24.5 kDa heterodimer, known as calprotectin (S100A8/A9 or MRP-8/14), which acts in either an autocrine or paracrine fashion and interacts with the receptor for toll-like receptor 4 (TLR4) and advanced glycosylation end-products (RAGE) [10]. The binding affinity of S100A9 for RAGE and TLR4 is significantly higher than that of S100A8/A9.

Furthermore, we examined the plasma concentration and transcriptional profile of S100A9 in circulating blood cells from AMI patients and controls. qRT-PCR was employed to validate the expression of S100A9 and its downstream genes in both groups at admission. The transcript levels of S100A9 in whole blood were correlated with left ventricle ejection fraction (LVEF) and MIRI events. Overall, our findings suggest that S100A9 plays a critical role in modulating cardiac inflammation and clinical outcomes during reperfusion, providing valuable insights for further experimental study.

## Materials and methods

### Data collection and download

Gene expression datasets GSE141512, GSE123342, and GSE109048, which include RNA-seq data from peripheral blood of 92 I/R samples and 47 control samples, were downloaded from the NCBI GEO database (https://www.ncbi.nlm.nih.gov/geo/). All datasets were derived from the GPL17586 platform of [HTA-2_0] Affymetrix Human Transcriptome Array 2.0 [transcript (gene) version]. For the I/R samples, patients underwent coronary angiography 0.5 to 12 h after disease onset, and blood samples for RNA profiling were obtained during revascularization. The datasets were merged using the ‘sva’ and ‘limma’ packages in R, with the batch effect removed by combat function. The analysis results were validated using the GSE48060 dataset.

### I/R-related genes and functional enrichment

Using the ‘limma’ package, 135 I/R-related differentially expressed genes (DEGs) were identified from the merged dataset, applying the cutoff criteria of|logFC| ≥ 0.5 and adj.P. Val < 0.05. Gene Ontology (GO) and Kyoto Encyclopedia of Genes and Genomes (KEGG) enrichment analyses were performed with the“clusterProfler” package. A protein-protein interaction n (PPI) network was constructed using STRING with a high confidence score of 0.7 and the results were analyzed and visualized using Cytoscape (V3.9.1). The degree topological algorithm in cytoHubba was used to identify the top 10 hub proteins based on degree centrality. To identify the final hub genes, we applied LASSO analysis via the ‘glmnet’ package in R. The diagnostic value was further validated using ROC curves with the GSE93272 dataset.

### Immune cells infiltration analysis

The “ssGSEA” package, which evaluates 13 immune function scores and 16 immune infiltration cell scores [11] in the “GSVA” R package, was employed to analyze immune infiltration in I/R. Spearman’s correlation was applied to assess the relationship between immune cell infiltration and the expression of hub genes.

### Study population and design

We prospectively enrolled 52 patients diagnosed with ST-elevation myocardial infarction (STEMI) within 12 h of symptom onset, in accordance with the current European Guidelines [12]. All patients underwent successfully primary percutaneous coronary intervention (PPCI) at Changshu NO.2 PEOPLE’s Hospital between January and April 2024. The control group consisted of 43 healthy individuals free of cardiovascular disease, matched to the I/R group by age and gender. Exclusion criteria for both groups included the following: older patients (> 85 years), pre-existing inflammatory disease, cardiomyopathy, Killip class IV, renal failure, previous myocardial infarction (MI) or revascularization, severe hepatic dysfunction, renal dysfunction, active infection, malignancy, or connective tissue disease. Upon discharge, all patients were treated according to current guidelines, receiving dual antiplatelet therapy, beta-blockers, and statins. Data on gender, age, body mass index, smoking history, previous medical history, and biochemical outcome were recorded. cTnT and NT-proBNP levels were assessed at admission, 12 h post-admission, and again 2–5 days later to identify peak values. Coronary lesion severity was quantified using the Gensini score. Echocardiography was performed one week after I/R, and LVEF was calculated using the Simpson method. All I/R patients were followed up for six months, with the incidence rates of major adverse cardiovascular events (MACEs) including myocardial reinfarction, emergent coronary revascularization, cardiac death, stroke, rehospitalization due to heart failure, and all-cause mortality reported. This study adhered to the principles outlined in the Declaration of Helsinki. The Ethical Committee of Changshu NO.2 PEOPLE’s Hospital approved the protocol (approval no. 2024-KY-SK09), and written informed consent was obtained from all participants.

### Definitions and classification of MIRI [12]

MIRI is defined as acute cardiac events occurring immediately after the reopening of the infarction-related artery (IRA). These events include slow flow, no reflow during percutaneous coronary intervention (PCI), hypotension (defined as blood pressure < 90/60 mmHg or systolic blood pressure (SBP) and/or diastolic blood pressure (DBP) decrease ≤ 30%), bradycardia, frequent ventricular contractions and other arrhythmias post-reopening. Additionally, MIRI may involve lethal ventricular arrhythmias requiring electrical cardioversion or new-onset heart failure.

### Blood collection and RNA extraction

Blood samples for RNA profiling were obtained from AMI patients using PAXgene™ RNA tubes before and 12–20 h after revascularization, typically in the morning (7:00 AM). Concurrently, venous peripheral blood samples for routine hematological (including leukocyte cell count and C-reactive protein levels) and biochemical tests were collected during hospital admission. For 16 AMI patients who underwent aspiration before PCI, intracoronary blood samples for RNA profiling were obtained using the Export (Medtronic) aspiration catheter after the lesion was crossed with a guidewire. Control samples, derived from individuals with coronary heart disease excluded based on the results of coronary angiography (CAG) results, were collected through venous blood sampling within one hour from medical examination. Blood samples for baseline hematological and biochemical assays were collected in standardized tubes containing dipotassium EDTA and processed within one hour of collection. The remaining blood was centrifuged at 2500 ×g for 10 min to isolate plasma. The resulting plasma was aliquoted and stored at -80 °C. Peripheral blood in PAXgene RNA tubes was incubated at room temperature for at least 2 h, then immediately transferred to the lab and stored at -80 °C until analysis.

### Mitochondrial Electron Transport Chain (ETC) complex I, Caspases-1 and IL-1β levels

Plasma levels of mitochondrial ETC complex I, Caspases-1 levels, and the inflammatory cytokines IL-1β were measured using an ELISA kit (LunChangShuo Biotech, Xiamen, China). The experiment was carried out according to the kit instructions precisely.

### Quantitative Real-Time PCR (qRT-PCR)

RNA was extracted from whole blood samples using Trizol^™^ reagent (ThermoFisher, Catalog No. 15596026) according to the manufacturer’s protocol, and mRNA was reverse-transcribed using ABScript Assessing S100A9 levels upon admission may guide the optimization of interventions in AMI patients, ultimately leading to improved clinical outcomes. III RT Master Mix (ABclonal, Catalog No. RK20429). The cDNA was then diluted five times with double-distilled water (ddH2O) and utilized for qPCR with Genious 2X SYBR Green. (Abclonal, Catalog No. RK21203). Real-time qPCR experiments were repeated three times using the Fast Real-time PCR 7500 System. The PCR amplification was carried out under the following conditions in a 10 µl volume: an initial denaturation at 95°C for 10 minutes, followed by 36 cycles of denaturation at 65°C for 5 minutes. The β-ACTIN gene was employed as an internal control, and relative expression levels were quantified using the 2-ΔΔCt method. The primers were as follows: S100A9:5’- GCTGGTGCGAAAAGATCTGC-3(forward) and 5’- GTGCATCTTCTCGTGGGAGG-3’ (reverse); TLR4: 5’- TGAGCAGTCGTGCTGGTATC-3’(forward) and 5’- TCGTCTCCAGAAGATGTGCC-3’(reverse); RAGE: 5’- GCTGGAATGGAAACTGAACACAGG-3’(forward) and 5’- TTCCCAGGAATCTGGTAGACACG-3’(reverse); NFKB: 5’- CTCCGAGACTTTCGAGGAAATAC-3’(forward) and 5’- GCCATTGTAGTTGGTAGCCTTCA-3’(reverse); NLRP3: 5’- TCTCACGCACCTTTACCTGC-3’(forward) and 5’- GATCCCAGCAGCAGTGTGAC-3’(reverse);β-Actin:5’- CGTGGACATCCGTAAAGACC-3’(forward) and 5’- AGTGCCAGCAGTTAATGTCAG − 3’ (reverse).

### Statistical analyses

Statistical analysis was performed using GraphPad Prism version 10.0 and R version 4.4.1. Continuous data are expressed as mean ± standard deviation, while categorical variables are presented as frequencies and percentages. Categorical variables were compared using the chi-squared test. For comparisons between two groups, t-tests were applied to normally distributed data, while the Wilcoxon matched-pairs signed-rank test was applied to paired, non-normally distributed data. The Mann-Whitney test was employed for unpaired data and non-normally distributed data. One-way ANOVA was performed for comparison among multiple groups and repeated measures ANOVA was applied for paired, normally distributed data, followed by Dunnett’s multiple comparisons. Spearman’s correlation was used to assess the association between continuous data. Multivariable logistic regression analyses were conducted to evaluate the capacity of genes to predict MIRI and MACES, adjusting for demographic and clinical parameters. A *p*-value of < 0.05 was considered statistically significant for all tests.

## Result

### Baseline characteristics

Table 1 presents the baseline characteristics of the study population. No statistically significant differences were observed between the I/R and control groups with respect to age, gender, BMI, hyperlipidemia, hypertension, smoking status, left atrium (LA), and serum biochemical levels, including eGFR creatinine and low-density lipoprotein. However, significant differences were observed in terms of diabetes, heart rate, LVEF, and serum levels of ALT, AST, and albumin. In the I/R group, CRP levels, white blood cell counts, and the percentage of neutrophils, lymphocytes, and eosinophils were significantly elevated.

**Table 1:** Baseline characteristics of the study population

	Control (43)	IR (52)	*P*
Age (years)	62.76 ± 10.82	62.50 ± 13.80	0.918
Sex (male), *n* (%)	32 (74.42%)	45 (86.54%)	0.134
Body-mass index (kg/m^2^)	23.90 ± 3.11	23.98 ± 2.77	0.889
Hypertension, *n* (%)	24 (55.81%)	30 (57.69%)	0.854
Diabetes, *n* (%)	5 (11.63%)	15(28.84%)	0.040
Smoking history, *n* (%)	9 (20.93%)	15 (28.84%)	0.377
Hyperlipidemia, *n* (%)	12 (27.91%)	9 (17.31%)	0.215
Heart rate (bpm)	69.14 ± 11.99	76.22 ± 9.48	0.012
Systolic pressure (mmHg)	134.95 ± 17.84	121.06 ± 16.96	0.185
ALT, U/L	18.67 ± 10.122	41.42 ± 28.72	0.000
AST, U/L	21.47 ± 9.91	172.42 ± 200.99	0.000
eGFR (ml/min)	94.15 ± 13.52	92.20 ± 18.96	0.150
Triglycerides (mmol/L)	1.93 ± 1.43	2.21 ± 2.70	0.481
Low-density lipoprotein (mmol/L)	2.71 ± 0.74	2.76 ± 0.88	0.466
Albumin(g/L)	41.40 ± 3.21	39.31 ± 3.93	0.006
Echocardiography measurements			
LAD, mm	39.26 ± 4.23	38.83 ± 4.81	0.649
LVEF, %	62.79 ± 4.29	57.39 ± 8.10	0.000
White blood cells on admission†			
Total white blood cells, 10^9^/L	6.54 ± 5.43	12.97 ± 16.86	0.018
Neutrophils, %	62.26 ± 9.65	75.61 ± 11.07	0.000
Lymphocytes, %	27.50 ± 8.37	17.50 ± 9.89	0.000
Monocytes, %	7.09 ± 3.99	5.73 ± 1.91	0.003
CRP, mg/L	6.20 ± 17.34	22.24 ± 32.32	0.003

Among the I/R patients, 16 individuals with thrombosis in myocardial infarction (TIMI) 0, 1, or 2-grade flow underwent aspiration and local tirofiban infusion. A total of 22 patients experienced MIRI-related complications following the intervention. These included 9 cases of no re-flow (including slow flow), 10 cases of malignant arrhythmia (comprising III atrioventricular block, ventricular tachycardia, and fibrillation, 2 cases occurring after no re-flow), 4 cases of hypotension and/or bradycardia (2 cases occurring post-no reflow), and 3 cases of new-onset heart failure. We categorized the I/R patients into MIRI and non-MIRI groups to explore the potential association between S100A9 expression and MIRI.

### Identification of DEGs in I/R

**Fig. 1:**
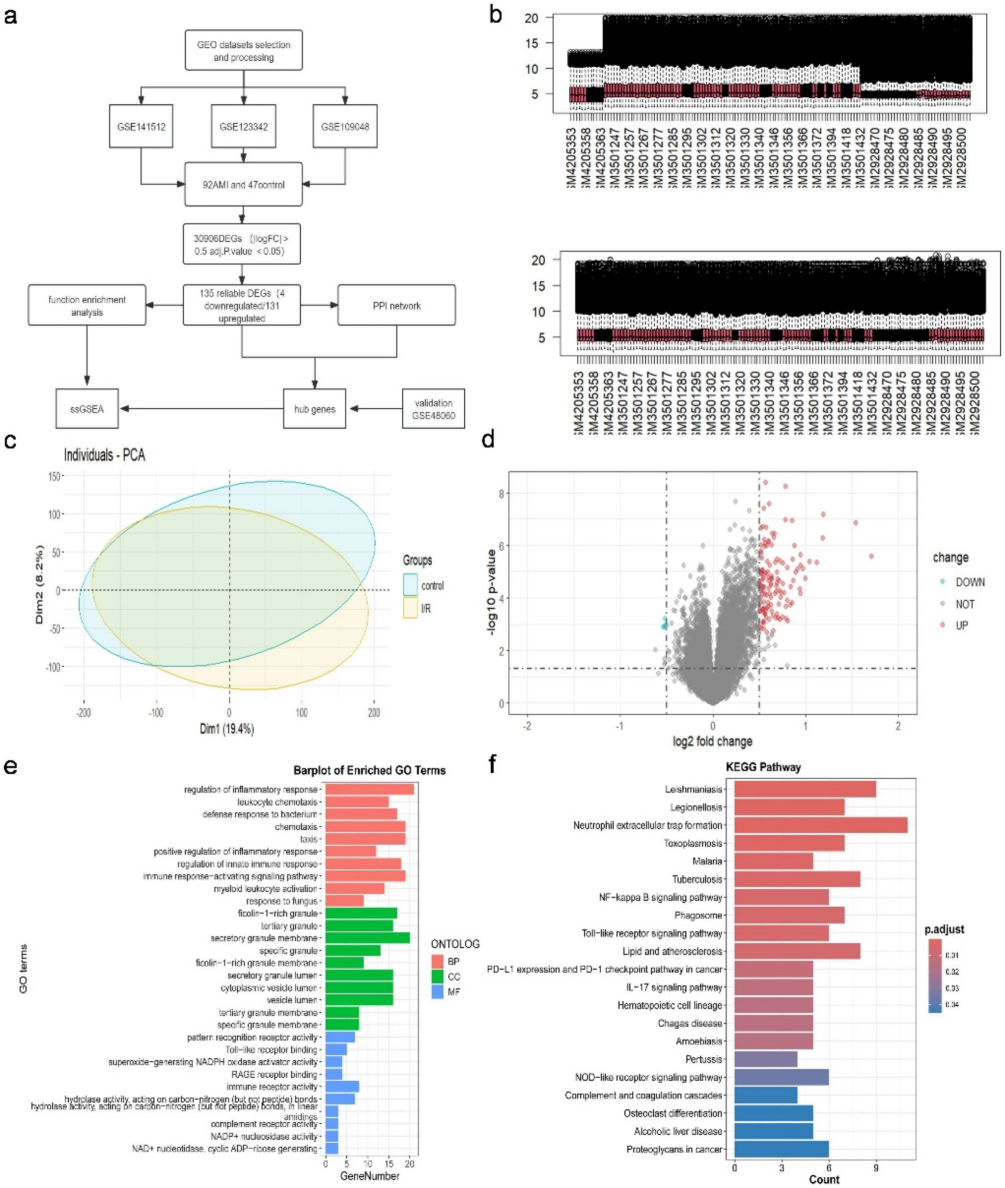
Identification of differentially expressed genes I/R-related genes and functional enrichment analysis. (**a**) Flow chart of the study. (**b**) Box plots for the expression levels of mRNAs before and after normalization by using the ComBat algorithm. (**c**) The results of the principal component analysis between IR and control group. (**d**) Volcano plot of DEGs constructed using the fold change values and P-adjust; red dots represent up-regulated differential genes, black dots represent nonsignificant genes, and green dots represent down-regulated differential genes. (**e**) GO term analysis and KEGG analysis of I/R differentially expressed genes. The significance of the gene gradually increases from blue to red

Differential expression analysis was performed on the merged dataset, which included 92 I/R samples and 47 control samples, using the ‘sva’ R package. The analysis workflow is shown in Fig. 1a. After removing the batch effect using the combat function (Fig. 1b), 135 I/R-related DEGs were identified, consisting of 131 upregulated and 4 downregulated genes. The Principal Component Analysis (PCA) and volcano plot of these DEGs are depicted in Fig. 1c and d. GO analysis (Fig. 1e) and KEGG analysis (Fig. 1f) were conducted to further characterize the I/R-related DEGs. The GO analysis encompassed three categories: biological process (BP), cellular component (CC), and molecular function (MF). In the BP category, DEGs were predominantly enriched in pathways associated with regulating inflammatory response, leukocyte chemotaxis, defense response to the bacterium, chemotaxis, taxis, etc. In the CC category, DEGs were linked to ficolin-1-rich granules, tertiary granules, secretory granule membranes, etc. In the MF category, DEGs were associated with pattern recognition receptor activity, Toll-like receptor binding, superoxide-generating NADPH oxidase activator activity, immune receptor activity, etc. The top 10 pathways identified by KEGG analysis included leishmaniasis, legionellosis, neutrophil extracellular trap formation, toxoplasmosis, malaria, tuberculosis, NFKB signaling pathway, phagosome, Toll-like receptor signaling pathway, and lipid and atherosclerosis.

### Screening and verification of hub genes

**Fig. 2:**
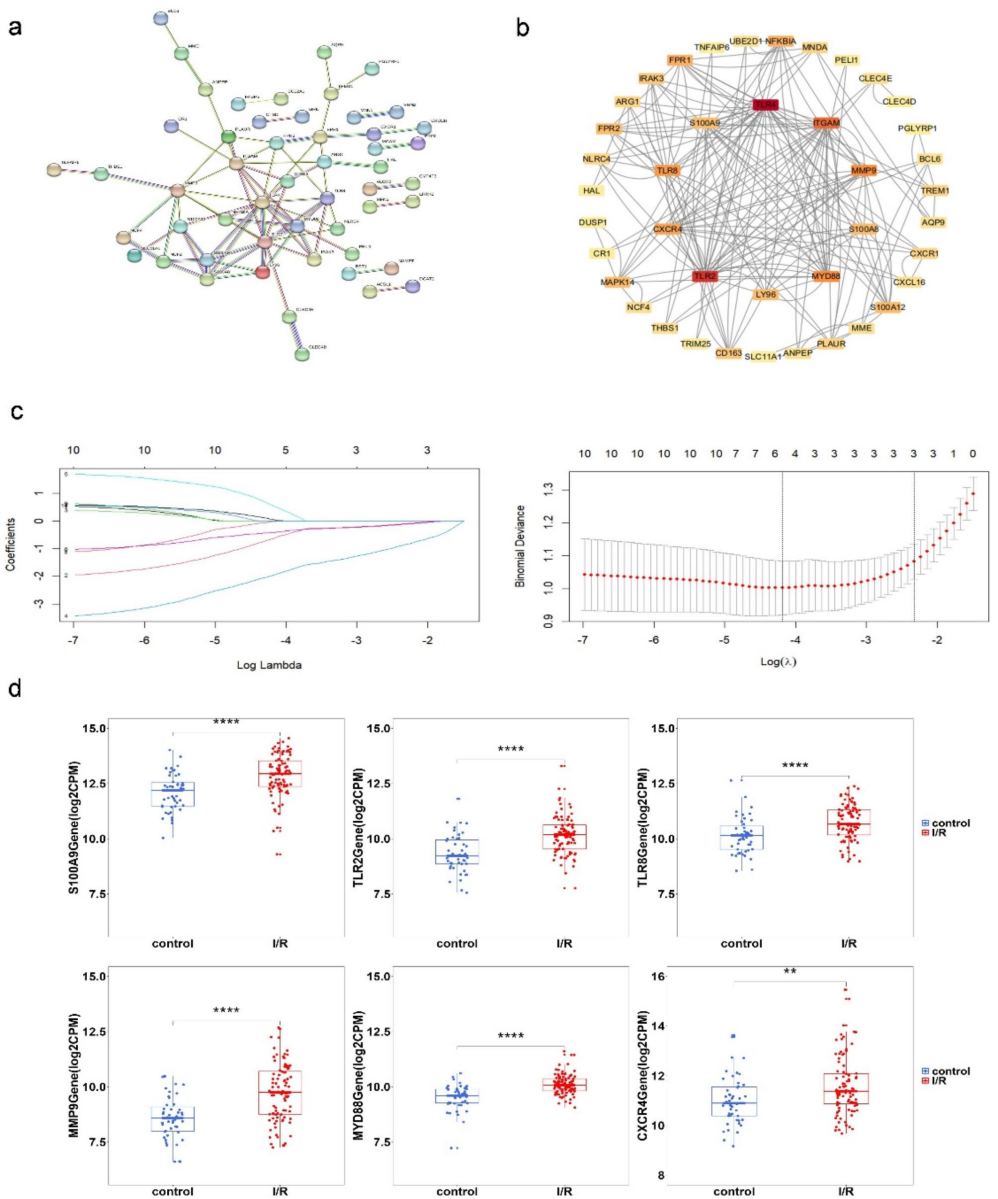
Screening novel regulator genes from DEGs. (**a**) PPI network analyzed by the STRING database. (**b**) Top 10 hub genes explored by CytoHubba. (**c**) LASSO coefficient profiles of the ten genes in IR. The log (lambda) sequence was used to construct a coefficient profile diagram. The LASSO model’s optimal parameter (lambda) was chosen. (**d**) The expression RNA level of novel regulator genes S100A9, TLR2, TLR8, MMP9, MYD88, and CXCR4 in the merged dataset between I/R(*n* = 92) and control(*n* = 47). * *P* < 0.05, **P < 0.01, ***P < 0.001, ****P < 0.0001

**Fig. 3:**
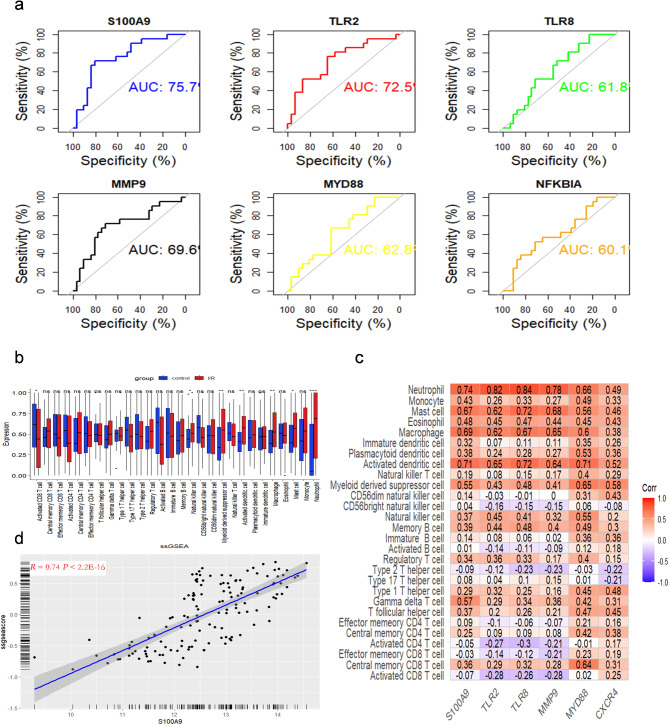
Correlation analysis between hub genes immune cell infiltration. (**a**) ROC assays for six genes based on GSE48060. (**b**) Distribution of 28 immune cells in control samples and I/R samples. (**c**) The associations between immune cell infiltration and six hub genes. (**d**) Correlation between S100A9 expression and Neutrophil content

A PPI network for the DEGs was constructed using the Retrieval of Interacting Genes/Proteins (STRING) and visualized in Cytoscape. As illustrated in Fig. 2a, the PPI network of shared genes consisted of 80 nodes and 121 edges. Using node degree centrality (NCC) analysis, the cytoHubba plugin identified the top 10 hub proteins: TLR4, TLR2, ITGAM, MYD88, TLR8, MMP9, CXCR4, S100A9, S100A8 and LY96 (Fig. 2b). To further refine the list of candidate gene, we employed the LASSO regression method, which narrowed the focus to six key regulatory gene for I/R (Fig. 2c). Distinct upregulation of S100A9, TLR2, TLR8, MMP9, MYD88 and CXCR4 was observed in I/R samples compared to control samples (Fig. 2d). To validate the expression patterns of these six genes, we performed additional analysis using the GSE46080 dataset. Receiver operating characteristic(ROC) curve analysis was conducted to assess the sensitivity and specificity of the six hub genes for I/R. S100A9 exhibited an AUC value > 0.75, indicating a strong diagnostic value, whereas the other genes showed an AUC value <0.75 (Fig. 3a). Our findings suggested that S100A9 may serve as a reliable biomarker for I/R.

### Evaluation and visualization of immune infiltration

The one-sample GSEA (ssGSEA) algorithm was employed to investigate differences in immune cell infiltration between I/R and control groups. Significant differences were observed in the distribution and proportions of various immune cell types, including Activated CD8 T cells, Natural killer cells, Macrophages, Activated dendritic cells, Myeloid-derived suppressor cells, Mast cells, and Neutrophils (Fig. 3b). Additionally, correlation analysis was performed between the expression of hub genes and the infiltration of 28 immune cells (Fig. 3c). In the I/R samples, S100A9 expression showed the strongest positive correlation with Neutrophil infiltration (Fig. 3d).

### Experimental validation

**Fig. 4:**
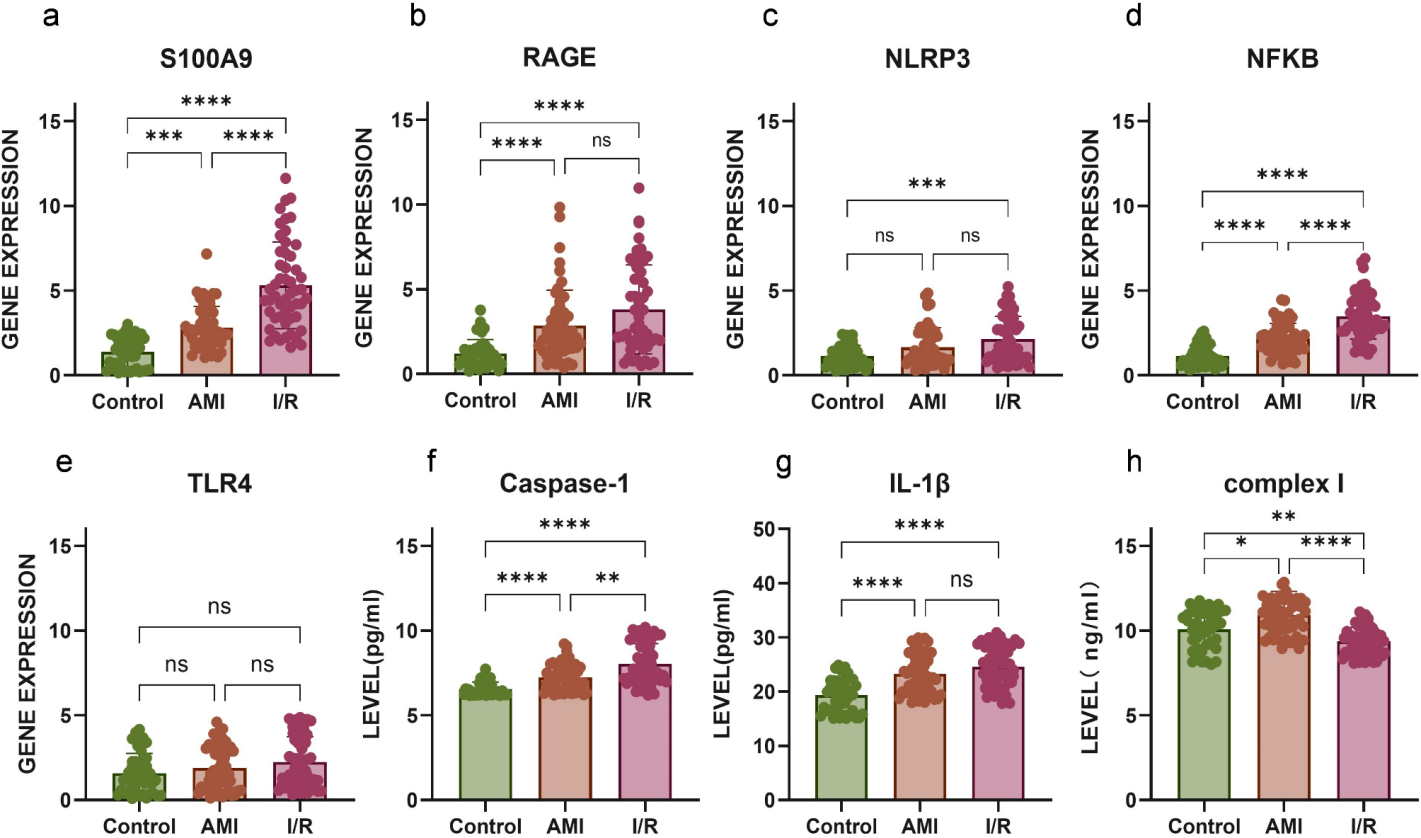
S100A9 signaling expression associated with AMI and I/R. (**a-e**) The mRNA expression levels of S100A9 (**a**), RAGE (**b**), NLRP3 (**c**), NFKB (**d**), and TLR4 (**e**) were measured both before and after PCI, in patients with AMI (*n* = 52) and compared to healthy controls (*n* = 43). (**f-h**) Plasma levels of mitochondria Caspases-1(**f**), IL-1β(**g**), ETC complex I(**h**) were also measured in patients with AMI(*n* = 52) and compared to healthy controls (*n* = 43). * *P* < 0.05, **P < 0.01, ***P < 0.001, ****P < 0.0001

We confirmed expression levels of S100A9 and its downstream signaling cascade using RT-qPCR and ELISA, confirming our previous finding from the DEG analysis. S100A9 expression was significantly higher in patients both before and after revascularization compared to the control group (Fig. 4a). Similarly, mRNA expression of RAGE (Fig. 4b)、NLRP3 (Fig. 4c), and NFKB (Fig. 4d) was significantly elevated in I/R patients. Moreover, S100A9 and NFKB expression levels were markedly increased in I/R versus AMI, with significant differences observed. Interestingly, TLR4 (Fig. 4e) expression showed similar trends to the other parameters but did not reach statistical significance between AMI, I/R, and control groups. The levels of mitochondrial ETC complex I, caspase-1, and the inflammatory cytokine IL-1β in plasma were also measured using ELISA. The results revealed that caspase-1 (Fig. 4f) and IL-1β (Fig. 4g) levels were elevated in the I/R group after reperfusion, while ETC complex I (Fig. 4h) expression was notably reduced compared to controls.

**Fig. 5:**
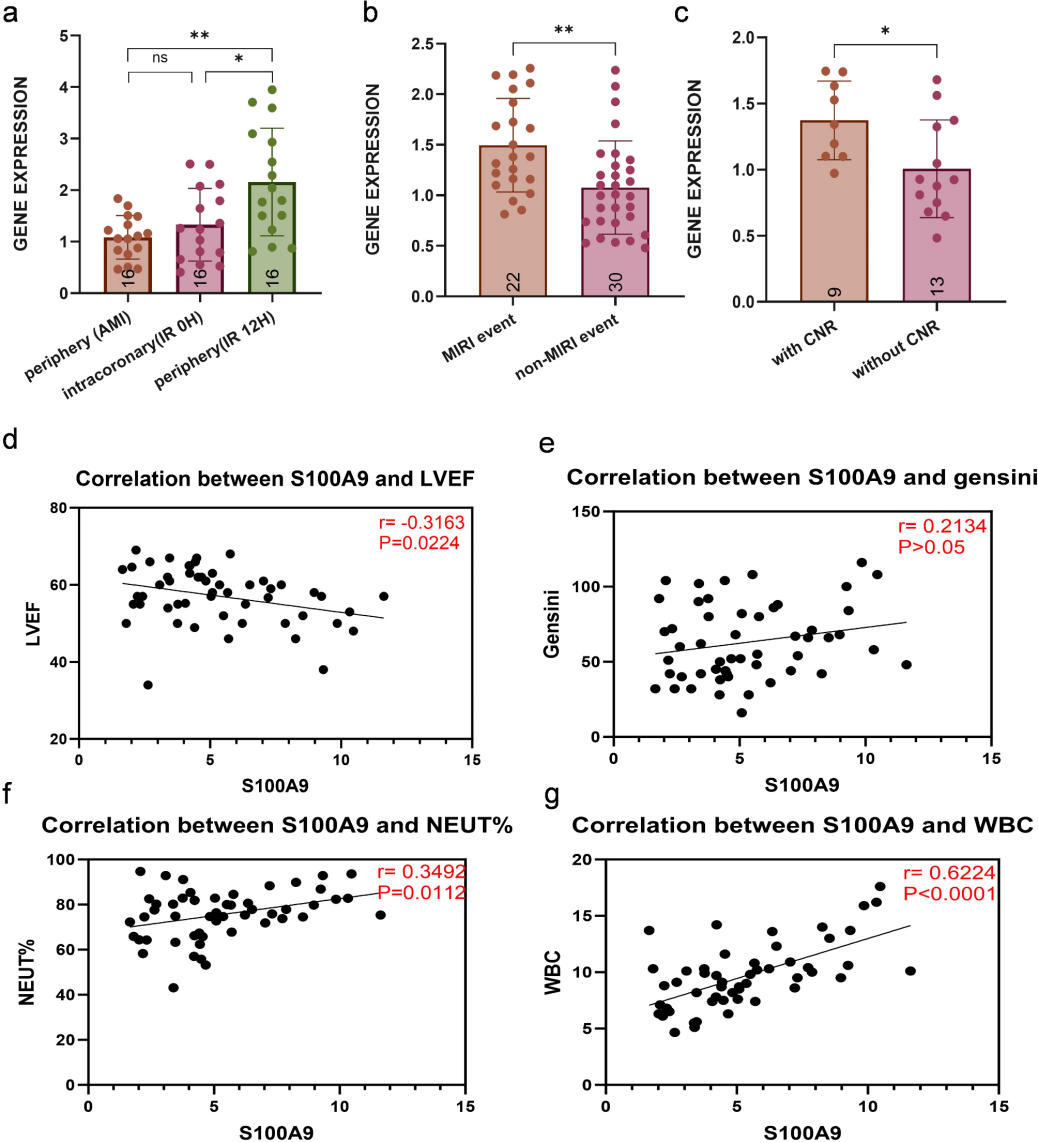
Correlation between S100A9 and cardiac events. (**a**) S100A9 expression was measured at admission before, 0, and 12 H after PCI in patients with AMI(*n* = 16). (**b**) S100A9 expression was upregulated in patients developing MIRI(*n* = 22) compared with those who did not(*n* = 30). (**c**) Among patients with MIRI, S100A9 expression was higher in CNR(*n* = 9) compared to those without CNR(*n* = 13). (d-g) Spearman Correlation in AMI patients Between Expression Levels of S100A9 and LVEF (**d**), gensini score (**e**), neutrophils percentage (**f**), and White blood cell counts (**g**). * *P* < 0.05, **P < 0.01, ***P < 0.001, ****P < 0.0001

Additionally, S100A9 expression was confirmed to be significantly higher in peripheral blood samples collected 12–20 h post-revascularization compared to intracoronary blood collected at 0 h. The expression of S100A9 showed no significant difference between AMI and 0 h I/R (Fig. 5a). Consistent with the I/R cohort, S100A9 transcript levels in whole blood were inversely correlated with LVEF (*r* = -0.3163, *p* < 0.05, Fig. 5d), positively correlated with the percentage of neutrophils (*r* = 0.3496, *p* < 0.05, Fig. 5f), and with white blood cell counts (*r* = 0.624, *P*<0.0001, Fig. 5g). However, no correlation was found between S100A9 levels and the Gensini score (*r* = 0.2134, *P*>0.05, Fig. 5e).

### Association of S100A9 with the MIRI and cardiovascular outcomes

We observed elevated S100A9 levels in patients with MIRI compared to those without MIRI (Fig. 5b). Of the 52 I/R patients, 22(42.3%) experienced MIRI complications, with the majority of these patients presenting with TIMI grade ≤ 2, which accounted for the largest proportion of MIRI cases. Our findings indicate that S100A9 expression is significantly higher in patients with TIMI grade ≤ 2 (including slow flow, no re-flow) compared with other MIRI patients (Fig. 5c).

**Fig. 6:**
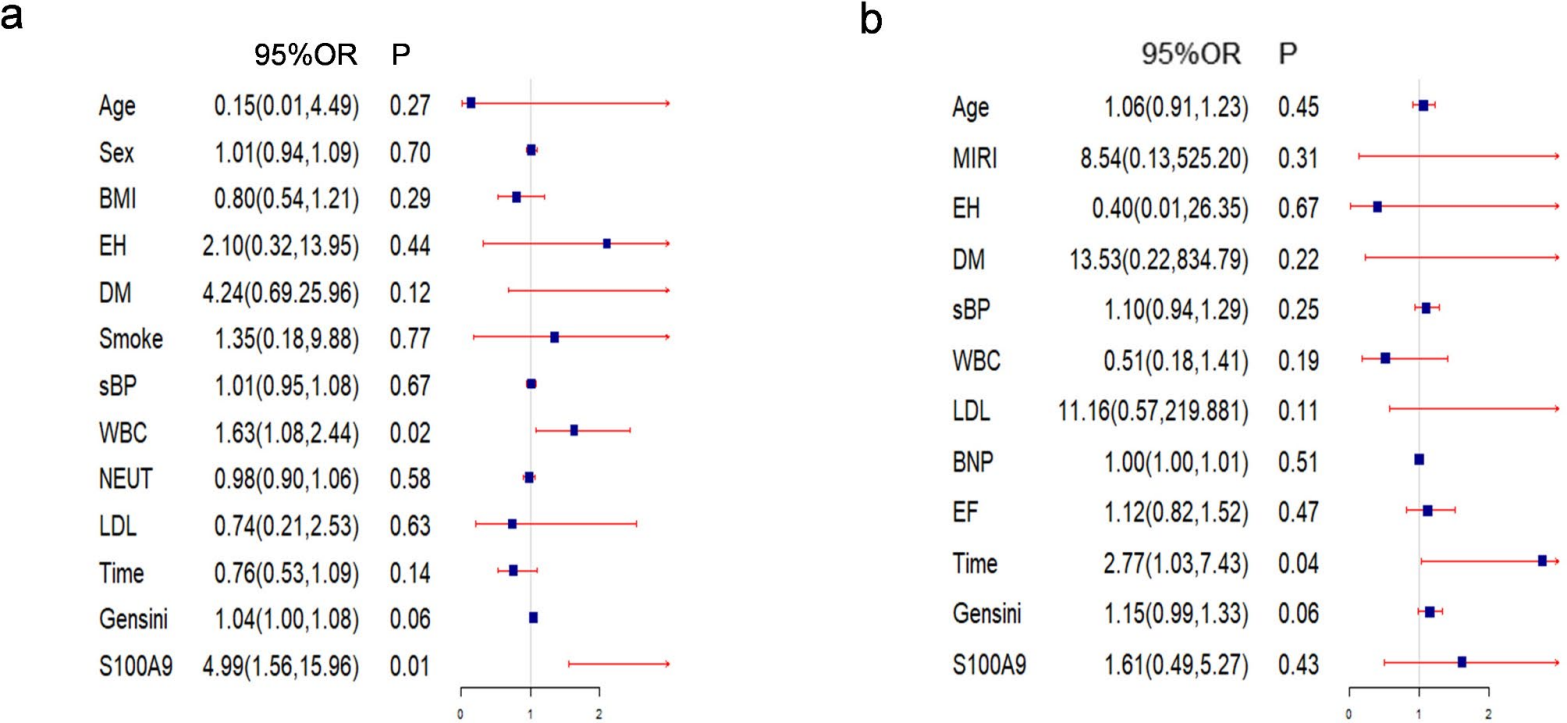
S100A9 is a predictor of MIRI in the validation cohort. The ability of S100A9 to predict MIRI (**a**), and MACEs (**b**), was determined using multivariable analyses

Logistic regression analysis revealed that S100A9 levels were univariate predictors of MACEs with odds ratios (ORs) of 4.99 (95% CI, 1.58, 15.96). Similarly, White blood cell counts were associated with MACEs, with ORs of 1.63(95% CI, 1.08, 2.44) (Fig. 6a).

Next, we examined the association between S100A9 RNA expression and the occurrence of MACEs. A cohort of 52 patients who underwent coronary interventions were followed for six months. Among them, one 62-year-old male patient experienced cardiac death, three patients had recurrent heart failure admissions, and one patient suffered upper gastrointestinal bleeding. Additionally, two patients experienced stenocardia and required resuscitation. The overall incidence of MACEs was 13.5%. Logistic regression analysis showed that the time of symptom onset was a univariate predictor of MACEs, with ORs of 2.77 (95% CI, 1.03, 7.43) (Fig. 6b). In contrast, S100A9 expression was not found to be a significant predictor of MACEs in this cohort.

## Discussion

MIRI is a common complication following reperfusion therapy in AMI, contributing to myocardial damage and influencing patient prognosis. Understanding the precise mechanisms of gene regulation involved in MIRI progression is essential for improving therapeutic interventions. However, most previous studies have been conducted at the animal and cellular levels, limiting their direct applicability to human disease.

In this study, we first identified 135 differentially expressed genes (DEGs) in I/R patients’ whole blood, of which 131 exhibited upregulation, suggesting a positive regulatory role in I/R progression. Next, we conducted GO and KEGG pathway enrichment analyses to identify functions and pathways associated with these DEGs. GO analysis revealed significant enrichment in the BP category for “regulation of inflammatory response” (*P*<0.001 ), and the top four terms of MF were enriched in “pattern recognition receptor ” (P<0.001), “Toll-like receptor binding” (P<0.001), “RAGE receptor binding” (P<0.001), superoxide-generating NADPH oxidase activator activity” (*P*<0.001), all of which are relevant to inflammatory responses and consistent with previous studies. I/R induces a sterile inflammatory response, characterized by significant neutrophil infiltration and the generation of inflammatory mediators, which exacerbates tissue injury and contributes to MIRI [13]. During the inflammatory response, necrotic and stressed/injured cells release DAMPs, which activate toll-like receptors (TLRs) [14] and the cell-surface RAGE [15], on both surviving parenchymal cells and infiltrating leukocytes. This activation triggers a cascade of inflammatory mediators, further amplifying the injury. GO function analysis revealed that the DEGs were significantly enriched in function associated with “superoxide-generating NADPH oxidase activator activity” (*P*<0.001), a significant source of reactive oxygen species (ROS) in cardiomyocytes and upregulated during I/R [16]. Utilizing PPI network analysis and the LASSO regression algorithm, we identified six potential critical biomarkers for I/R: S100A9, TLR2, TLR8, MMP9, MYD88, and CXCR4. We further validated the diagnostic potential of these biomarkers using the GSE48060 dataset, with S100A9 emerging as a critical biomarker for I/R through ROC analysis. Previous research has shown that S100A9 plays a crucial role in inflammatory and reparative immune responses following myocardial infarction across various models [17]. However, its role in human I/R has not been well explored.

In addition to bioinformatics analysis, we examined immune cell infiltration in I/R samples using the ssGSEA algorithm. This approach allowed us to analyze the infiltration of 28 immune cell types in I/R samples. Our results revealed that I/R patients exhibited significantly higher levels of neutrophil infiltration compared to controls. Clinical blood tests further corroborated these findings, showing elevated white blood cell and neutrophil percentages in I/R patients. Neutrophils are rapidly recruited to the injured heart following reperfusion, acting as primary and secondary mediators of lethal injury to coronary vascular endothelium and cardiomyocytes [18]. Single-cell RNA sequencing (scRNA-seq) analysis [19] revealed distinct functional roles for cardiac neutrophil subsets in both MIRI and MI. Neutrophils in MIRI peaked on the first day and quickly returned to baseline, occurring much earlier than those in MI. Importantly, our KEGG data support early reports indicating that activated neutrophils release neutrophil extracellular traps (NETs) during I/R injury, contributing to myocardial no-reflow [20]. The NETs interact with red blood cells and platelets to form plugs, which are carried by blood flow into microvessels, resulting in embolisms that obstruct tissue perfusion even after large coronary arteries are restored. This obstruction can lead to myocardial infarction and early heart failure [21, 22]. Emerging evidence [23] suggests that neutrophils also play a role in cardiac repair by promoting macrophage polarization towards a reparative phenotype three days after post-infarction. However, clinical trials have demonstrated that anti-neutrophil therapy has no salubrious effects in the context of reperfusion injury [18]. Thus, anti-neutrophil interventions may not be effective in minimizing reperfusion-related injury.

Moreover, S100A9 was found to be strongly correlated with neutrophil infiltration in both bioinformatics and clinical data. This finding supports the hypothesis that S100A9 is primarily released by neutrophils, especially in the context of myocardial injury. Previous studies have shown that S100A9 is present at low levels in neutrophils under normal conditions but is released during inflammation or cell death [24]. In Mass’s study, infiltrating CXCR4^+^ neutrophils was identified as the primary source of S100A9 in the tumor microenvironment [25]. Coincidentally, CXCR4 was one of our bioinformatics hub genes. In the infarcted myocardium, the alarmin S100A9 is released, where it binds to TLR4 or the RAGE receptors, subsequently activating the NFKB [26] and NLRP3 [27]. This process highlights the pivotal role of neutrophils in the pathogenesis of lethal myocardial injury. Additionally, research has shown that E-selectin mediates the formation of N-terminal gasdermin D pores in neutrophils in an NLRP3-dependent manner, which leads to the release of S100A9 [28]. Upon release, S100A9 enhances the expression of inflammatory cytokines such as IL-1β, IL-18, and TNF-α, along with chemokines, and fibrosis markers. It also stimulates fibroblast proliferation, thereby activating the inflammation response and promoting tissue fibrosis. These pathophysiological changes interact with various cell types—including endothelial cells, monocytes, macrophages, platelets, and cardiomyocytes— to mediate cardiomyocyte death, contributing to lethal MIRI, characterized by mitochondrial dysfunction, oxidative stress, and inflammation [29].

Lastly, we performed RT-qPCR to verify elevated expressions of S100A9 and its downstream signaling cascade in AMI patients who received coronary interventions. Our study did not find a significant difference in TLR4 expression, which may be attributed to the fact that the samples were sourced from peripheral blood. Prabhakara R et al. cultured BM progenitor cells isolated from WT, Rage−/−, and Myd88−/− mice in response to S100A9, and only the Rage−/− cells did not show increased proliferation [32]. They proposed that S100A9 engages with RAGE on bone marrow progenitor cells to enhance their proliferation. Consistent with the bioinformatics analysis, our experiment results suggest that impaired macrophage phenotypic shift, due to the absence of neutrophil secretome, leads to inefficient clearance of dead cells, contributing to a dysregulated healing response.

This study demonstrated dynamic changes in whole blood S100A9 expression in AMI patients before and 12–20 h after PCI, as well as versus control. Li et al. proved that the S100A9 level peaked one day post-PCI [29]. However, we did not detect a significant difference in S100A9 expression between AMI and 0 h after PCI. This discrepancy may partly arise from variations in blood sources. A previous study [30] emphasized the primary role of S100A8/9 and their downstream mediators (e.g. NLRP3) in shaping the inflammatory response following myocardial infarction. Given the increased expression of S100A9 and its downstream signaling cascade in MIRI, we speculated that the injury caused by S100A9 signaling is more severe in MIRI.

Furthermore, we observed higher serum protein expression of caspase-1 and IL-1β levels in I/R. We also detected inhibition of ETC complex I protein expression in I/R, which resulted in defects in mitochondrial ATP bioenergetics and imbalances of NAD^+^/NADH ratio. These findings suggest that S100A9 may activate the inflammatory response and mitochondrial dysfunction by modulating specific signaling pathways in I/R patients, positioning it as a master regulator in both I/R and MIRI. Previous studies have emphasized that elevated plasma levels of S100A9 post-PCI correlate with longer hospital stays and a higher incidence of heart failure in AMI patients [31]. Marinković et al. found a correlation between elevated plasma S100A8/A9 levels during acute events and reduced LVEF [32]. Consistent with this study, our data suggested that the RNA expression of S100A9 was related to LVEF seven days post-AMI.

In PPCI for STEMI patients, opening an infarct-related artery inevitably leads to reperfusion injury, which can cause myocardial stunning, reperfusion arrhythmia, and the slow-flow or no-reflow phenomenon. Currently, there is no clinical method available to effectively mitigate myocardial injury resulting from reperfusion. This study assessed the extent of MIRI in STEMI patients undergoing PPCI, based on their clinical characteristics and coronary angiography findings.

At admission, patients with MIRI exhibited significantly elevated whole blood S100A9 RNA expression compared to those without MIRI. In vivo, the influence of S100A9 in mitochondrial dysfunction and cardiomyocyte death was confirmed in the MIRI mouse model. Single-cell RNA sequencing has also shown that S100A9 facilitates the shift from acute inflammation to fibrotic remodeling following I/R [27]. Additionally, our study suggested that each 1-unit rise in the S100A9 index independently correlates with an increased risk of MIRI events. However, the predictive role of the S100A9 for MACEs in AMI remains unclear due to the limited follow-up period.

According to the statistics, the coronary no-reflow phenomenon (including slow flow) is the primary MIRI event, occurring in up to 17.3% of our patients, although it is less frequent compared to other outcomes [33]. Our data also indicated that S100A9 expression was elevated in patients with no reflow. Coronary artery no-reflow (CNR) [34] following AMI is primarily a reperfusion phenomenon after PCI, often referred to as microvascular obstruction (MVO), which manifests clinically as coronary TIMI grades 0, 1and 2 flow after ruling out mechanical causes like coronary spasm and occlusion. Bates reported the occurrence of coronary no-reflow through angiographic contrast density in 1986 [35]. No flow is more frequently identified by angiographic visualization following the onset of primary revascularization. Predicting and preventing CNR is crucial for improving the efficacy of coronary interventions, as its incidence and extent are linked to adverse clinical outcomes such as increased in-hospital mortality, reinfarction rates, heart failure, and cardiogenic shock [36]. However, the mechanisms of CNR remain not fully understood. Existing theories, supported by clinical settings and animal models [37], suggest that microvascular circulation alterations caused by vasospasm, endothelial swelling, neutrophils plugging, activation of inflammatory pathways, and cellular edema contribute to the development of CNR. Patients with CNR exhibited significantly higher white blood cell and neutrophil counts [38]. Su et al. demonstrated that the inflammatory response in CNR activates the NLRP3 inflammasome and promotes the inflammatory cascade. However, the underlying mechanisms of neutrophil infiltration and increased chemokine expression that drive CNR development are yet to be fully elucidated. Taken together, our data suggest that active neutrophils exacerbate the inflammatory response by releasing S100A9 and contribute to CNR after I/R. Therefore, S100A9 may represent a new therapeutic target to prevent CNR in AMI patients undergoing reperfusion. Further experiments involving mechanisms are needed to confirm the effect of S100A9 on the CNR phenomenon. Our analysis of TIMI flow grade consistently showed no-reflow during the primary procedure, which demonstrates that TIMI flow assessment has limited sensitivity in identifying no-reflow [39]. Other invasive approaches, such as myocardial blush grade and computer-assisted myocardial blush quantification, may prolong door-to-balloon time and should be avoided in STEMI patients. In this context, the association between S100A9 and CNR offers clinical evidence for using S100A9 as a predictor in AMI patients prior to PCI. Surgeons may use this information to take immediate preventive measures, such as thrombus aspiration, primary stenting, and avoiding high-pressure inflation, which may ameliorate the degree of no-reflow [40] before the intervention.

### Study limitations

Our results should be interpreted with consideration of several limitations. First, this study’s single-center design, small sample size, and short follow-up period may introduce bias into the data. Additionally, echocardiographic follow-up data was incomplete for some patients. Second, blood samples were collected 12–20 h post-reperfusion during the acute phase of MI. Due to the time-dependent nature of RNA profiling, variations in RNA profiles at subsequent time points could not be assessed. Third, there was an imbalance in medication use between MI patients who developed MIRI and those who did not. Lastly, while we demonstrated the expression of S100A9 signaling in blood cells, evidence regarding its effects on cardiomyocytes and endothelial cells is lacking.

## Conclusion

This study identifies S100A9 as a novel regulator in I/R through bioinformatics analysis. Whole blood, used as a surrogate tissue provides insights into S100A9 upregulated NFKB/NLRP3 inflammasome activations (caspase-1 and IL-1β) in I/R patients. Furthermore, we demonstrate that elevated S100a9 levels post-PCI predict the incidence of MIRI, particularly CNR, in patients with AMI. Our findings highlight S100A9 signaling as a potential therapeutic target for mitigating MIR injury. Assessing S100A9 levels upon admission may guide the optimization of interventions in AMI patients, ultimately leading to improved clinical outcomes.

## Data Availability

No datasets were generated or analysed during the current study.
